# Myocardial protection by adenosine triphosphate−sensitive potassium channel opener diazoxide involves sulfonylurea receptor 2 subunit

**DOI:** 10.1016/j.xjon.2025.10.018

**Published:** 2025-10-30

**Authors:** Jie Wang, AlleaBelle Bradshaw, Robert Tryon, Rachel Pan, Sari D. Holmes, Colin Nichols, Jennifer S. Lawton

**Affiliations:** a Division of Cardiac Surgery, Department of Surgery, Johns Hopkins University School of Medicine, Baltimore, Md; b Department of Cell Biology and Physiology, Washington University School of Medicine, St Louis, Mo

**Keywords:** cardioprotection, diazoxide, Langendorff, cardiac surgery, sulfonylurea receptor

## Abstract

**Objective:**

Pharmacologic openers of adenosine triphosphate-sensitive potassium (K_ATP_) channels mimic ischemic preconditioning and are cardioprotective. The components of any relevant channel have not been identified, although several candidate protein targets have been proposed. Identification of implicated K_ATP_ channel components would allow directed drug targeting for clinical trials. We have investigated various channel subunits (Kir1.1, SUR1, Kir6.1, Kir6.2) as candidates, but none has been implicated. To complete our evaluation of recognized K_ATP_ channel components, we examined the potential role of the regulatory sulfonylurea receptor SUR2 in diazoxide (DZX) cardioprotection in a model of prolonged global myocardial ischemia.

**Methods:**

Mice lacking SUR2 (knockout) and wild-type litter mates (genotype confirmed) were randomly assigned to 90 minutes of global ischemia in a Langendorff model after hypothermic hyperkalemic cardioplegia with or without DZX (100 μM/L) (N = 9-14 per group). Left ventricular developed pressure, end-diastolic pressure, and coronary flow were compared before and after global ischemia. Data acquisition and interpretation were blinded.

**Results:**

Prolonged global ischemia with cardioplegia was associated with reduced left ventricular developed pressure and increased end-diastolic pressure that was prevented by DZX in wild-type but not SUR2 knockout hearts, consistent with DZX cardioprotection being mediated by a K_ATP_ channel with a SUR2 component.

**Conclusions:**

Cardioprotection provided by DZX was lost with genetic deletion of SUR2, implicating SUR2 as a subunit of a cardioprotective K_ATP_ channel. Identification of the subunits of a cardioprotective K_ATP_ channel would allow targeted pharmacologic therapy to reduce myocardial stunning after global ischemia during cardiac surgery.

## Graphical Abstract

**Figure:**
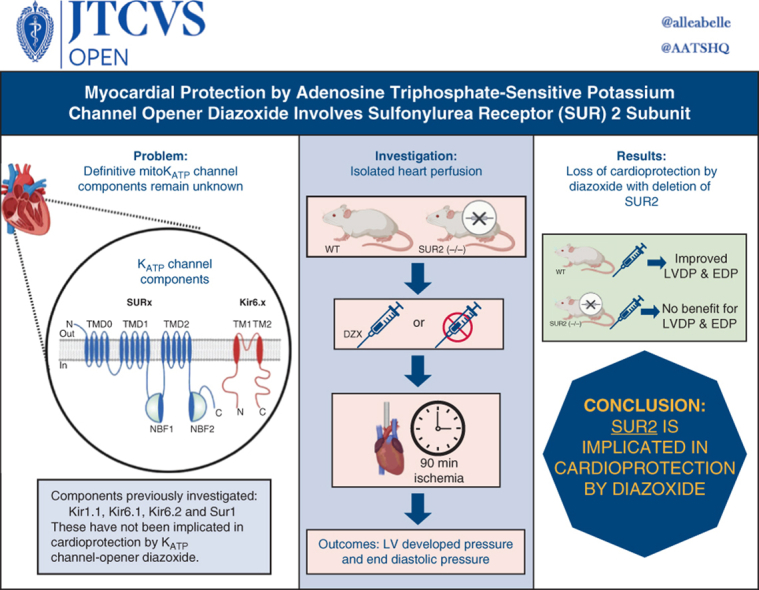

**Figure:**
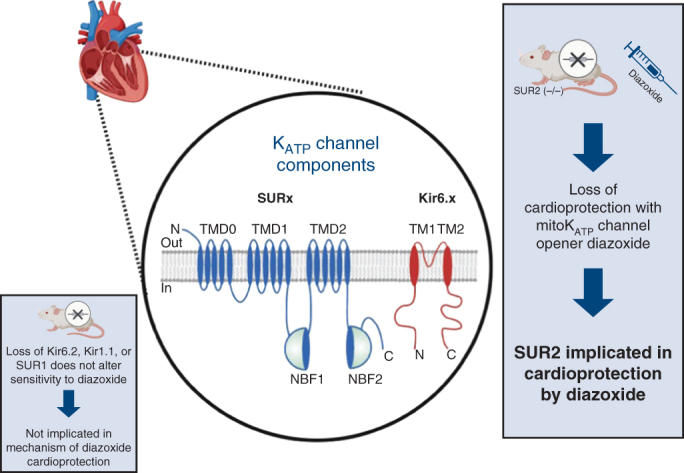
Components of a diazoxide-responsive K_ATP_ channel are unknown. This study implicates SUR2.

**Central Message**

Cardioprotection provided by the K_ATP_ channel opener diazoxide was lost with genetic deletion of SUR2, suggesting SUR2 may be a subunit of a cardioprotective K_ATP_ channel.

**Perspective**

Pharmacologic ischemic preconditioning agents such as the K_ATP_ channel opener diazoxide (DZX) provide myocardial protection in multiple models, but the molecular mechanism of action remains unknown. Mice lacking SUR2 demonstrate a loss of myocardial protection by DZX, implicating SUR2 in DZX cardioprotection.

There is a growing need for improved strategies to protect the heart from ischemia and reperfusion injury in cardiac surgery.^1^, ^2^, ^3^ A lack of adequate myocardial protection leads to myocardial stunning, which is a reversible myocardial injury that is associated with adverse outcomes after surgery.^4^^,^^5^ Pharmacologic openers of adenosine triphosphate-sensitive potassium (K_ATP_) channels mimic ischemic preconditioning and are cardioprotective.^6^ Diazoxide (DZX) is a K_ATP_ channel opener that acts on multiple K_ATP_ channel subtypes and can reduce myocardial stunning after cardiac surgery.^7^, ^8^, ^9^, ^10^, ^11^, ^12^ We have begun a phase 1 safety and feasibility clinical trial approved by the Food and Drug Administration to evaluate DZX in cardioplegia in humans (clinicaltrials.gov NCT06308107) to examine this potential benefit.

K_ATP_ channels are inwardly rectifying potassium channels composed of 4 pore-forming (Kir) subunits and 4 regulatory (sulfonylurea receptor [SUR]) subunits.^13^^,^^14^ Identification of K_ATP_ channel components involved in DZX protection would lead to a better understanding of the mechanism of action and the ability to target the channel for clinical trials.^15^ We have investigated various channel subunits as candidates, including Kir1.1, Kir6.1, Kir6.2, and SUR1 (Table 1).^10^^,^^16^, ^17^, ^18^, ^19^, ^20^, ^21^, ^22^ However, none has been implicated in cardioprotection by DZX. SUR2 is the only remaining known K_ATP_ channel component that is yet to be evaluated. The role of this subunit in cardioprotection by DZX remains unknown.^23^, ^24^, ^25^, ^26^

**Table 1:** Summary of results using K_ATP_ channel subunit genetic deletion, gain of function, or pharmacologic inhibition in mouse models to localize site of action of cardioprotection by diazoxide

Mouse strain or channel inhibitor	Model		
	Mitochondria	Myocyte	Langendorff
Kir subunits			
Kir6.1 KO		Increased tolerance to stress, responsive to DZX^16^	
Kir6.1 GOF		Increased tolerance to stress, responsive to DZX^17^	
Kir6.2 KO		KO *does not swell* in response to CPG∗^18^ KO *does not swell* in response to MI; therefore, cannot assess effect of DZX†^19^	
Kir1.1 (ROMK) cardiac-specific KO			Kir1.1 KO responsive to DZX‡^20^
Kir1.1 inhibition by VU591			DZX cardioprotective in presence of VU591‡^20^
Kir1.1 inhibition by TpnQ	No change in mitochondrial volume (mitochondrial volume change is proposed surrogate for K_ATP_ activity)^21^	TpnQ blocks DZX cardioprotection†^21^	
SUR subunits			
SUR1 KO	DZX inhibits SDH in SUR1 KO mitochondria§^22^	SUR1 KO eliminates DZX cardioprotection§^10^	SUR1 KO responsive to DZX§^20^
SUR2 KO			SUR2 not responsive to DZX (Wang et al, in preparation)

Techniques to localize the site of action of cardioprotection by DZX have included genetic deletion, GOF, or pharmacologic inhibition of specific subunits. VU591 is a Kir1.1 inhibitor. TpnQ is a Kir subunit inhibitor. *KO*, Knockout; *DZX*, diazoxide; *GOF*, gain-of-function; *CPG*, cardioplegia; *MI*, metabolic inhibition; *ROMK*, renal outer medullary potassium; *TpnQ*, Tertiapin Q; *K*_*ATP*_, adenosine triphosphate-sensitive potassium; *SUR*, sulfonylurea receptor; *SDH*, succinate dehydrogenase.

∗ Kir6.2 KO does not swell with exposure to hypothermic, hyperkalemic CPG.

† Kir6.2 KO does not swell with exposure to metabolic inhibition.

‡ Results between myocyte and Langendorff models for Kir1.1 are conflicting.

§ Results between mitochondrial, myocyte, and Langendorff models for SUR1 are conflicting, suggesting different mechanisms in each model.

We have used mice lacking known K_ATP_ channel subunits to clarify the molecular mechanism of action of DZX.^10^^,^^27^ To complete the investigation of known K_ATP_ channel components, we hypothesized that SUR2 would be implicated in DZX cardioprotection in our established model of prolonged global myocardial ischemia (Figure 1).

**Figure 1:**
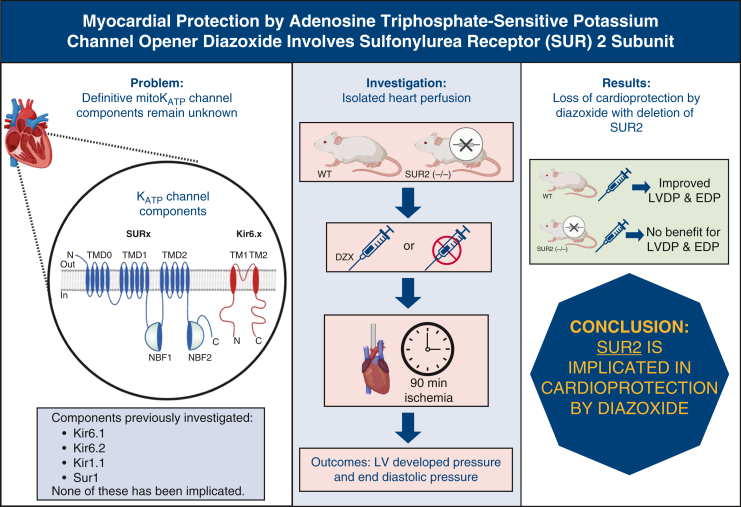
Components of a diazoxide-responsive cardioprotective K_ATP_ channel subunit remain unknown. Wild type (WT) and SUR2 knockout hearts were used to investigate the role of SUR2 in cardioprotection by diazoxide. Unlike WT hearts, hearts lacking SUR2 did not have improved LVDP and reduced EDP in response to DZX. Figure created using BioRender. *K_ATP_*, Adenosine triphosphate-sensitive potassium; *SUR*, sulfonylurea receptor; *LVDP*, left ventricular developed pressure; *EDP*, end-diastolic pressure; *DZX*, diazoxide.

## Methods

All animal procedures were approved by the Animal Care and Use Committee at Johns Hopkins University (protocol MO19M369, approved September 25, 2022). Animals received humane treatment in abidance with the principles of laboratory animal care as stated by the National Society for Medical Research. Although both sexes of animals were used in all experiments, subgroup analyses of results by sex were not performed because of the lack of power. No consent was needed because no human subjects were included.

CRISPR/Cas9 gene editing was used to introduce the equivalent of the human SUR2 [R1154Q] mutation into the mouse *ABCC9* gene.^28^ Homozygous SUR2 KO mice and C57BL/J wild-type littermates genotype was verified by Transnetyx (Cordova).

### Isolated Heart Preparation

Mice (SUR2 KO or WT C57BL/J [SUR2 KO littermate], either sex) underwent rapid cardiectomy as previously described.^7^^,^^29^ Hearts were submersed in ice-cold Krebs-Henseleit buffer (KHB) during aortic cannulation. Hearts were excluded if aortic cannulation time exceeded 5 minutes. Hearts were perfused in a Langendorff fashion (retrograde via the aorta) with KHB (118.5 mmol/L NaCl, 25.0 mmol/L NaHCO_3_, 3.2 mmol/L KCl, 1.2 mmol/L KH_2_PO_4_, 1.2 mmol/L MgSO_4_, 1.4 CaCl_2_, and 5.5 mmol/L D-glucose). A balloon (custom-made in our laboratory) was introduced into the left ventricle (LV) and connected to a pressure transducer (HP1290 C; ADInstruments) and an amplifier (20-4; Hugo Sachs Elektronik-Harvard Apparatus). Hearts were epicardially paced at 450 beats per minute and surrounded by a warm (37 °C) water-jacketed beaker. Coronary perfusion pressure was maintained at 80 mm Hg (the zone of autoregulation for coronary vasculature in murine hearts) by Langendorff column height.

### Experimental Protocol

**Figure 2:**
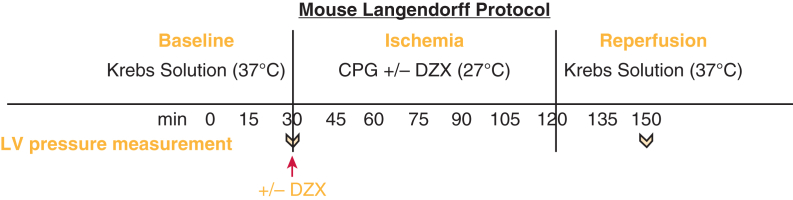
Isolated mouse heart (Langendorff) model of global ischemia. Isolated mouse hearts (SUR2 knockout, or wild-type SUR2 knockout littermates) underwent Langendorff baseline perfusion with Krebs-Henseleit buffer (KHB) solution for 30 minutes followed by arrest (*red arrow*), and global ischemia (90 minutes) with test solution (hypothermic hyperkalemic cardioplegia ± K_ATP_ channel opener diazoxide), and then reperfusion with KHB for 30 minutes. Left ventricular pressures were recorded during baseline (at 30 minutes) and after 30 minutes (time 150) of reperfusion at identical balloon volumes (*yellow symbols*). Permission obtained from Elsevier, license 5994820944601 (3/23/2025). *SUR*, Sulfonylurea receptor; *K_ATP_*, adenosine triphosphate-sensitive potassium; *CPG*, cardioplegia; *DZX*, diazoxide.

Balloon volume was adjusted to an end-diastolic pressure (EDP) of 2.5 mm Hg. During a 30-minute baseline period, LV pressure was measured at increasing 1.4-μL balloon volume increments for baseline data collection (Figure 2). The LV balloon volume was then returned to the initial volume, and the heart was arrested for 90 minutes using the test solution.

Randomly assigned test solutions included hyperkalemic cardioplegia (CPG: Plegisol; 110 mmol/L NaCl, 10 mmol/L NaHCO_3_, 16 mmol/L KCl, 32 mmol/L Mg, and 2.4 mmol/L CaCl_2_, titrated to pH 7.8 with 8.4% NaHCO_3_ solution) or CPG + DZX 100 μmol/L (n = 9-14). After 90 minutes of global ischemia, KHB retrograde perfusion and epicardial pacing were resumed. After 30 minutes, LV pressures were recorded at the identical balloon volumes used during the baseline period as previously described.^7^^,^^29^^,^^30^ Investigators were blinded to the test group during experiments and data analysis. DZX was administered before ischemia because it is cardioprotective only when administered before stress.^31^

### Data Acquisition and Analysis

LV EDP and left ventricular developed pressure (LVDP) were determined from digitalized data files using LabVIEW 2014 (National Instruments) and compared over a series of identical intracavitary balloon volumes. Coronary flow rates were measured at baseline and every 5 minutes after reperfusion by an inline N-series flow probe and a T206 flow meter (Transonic Systems) and compared, as described previously.^7^

### Statistical Analysis

Coronary flow between groups was examined using repeated measures analysis of variance (ANOVA). Changes in EDP and LVDP from baseline by balloon volume and mouse type groups were examined using repeated measures ANOVA with Huynh-Feldt correction when the assumption of sphericity was violated. The results of these analyses provided the main effect of balloon volume (regardless of group), the main effect of mouse type groups (collapsed across balloon volumes), and the interaction of balloon volume and group to indicate if the pattern of results over different balloon volumes differed by group. Pairwise comparisons between the groups at individual balloon volumes were conducted using independent-samples *t* tests when indicated on the basis of the results of the repeated measures ANOVAs. Sample size calculations were based on previous work in this model. Studies were designed to detect a 5% ± 4% difference using an α = 0.05 and a power of 0.80. Analyses were conducted with IBM SPSS, Version 28.0 (IBM Corp).

## Results

### LV Developed Pressure and EDP Before and After Global Ischemia

#### LV developed pressure

**Figure 3:**
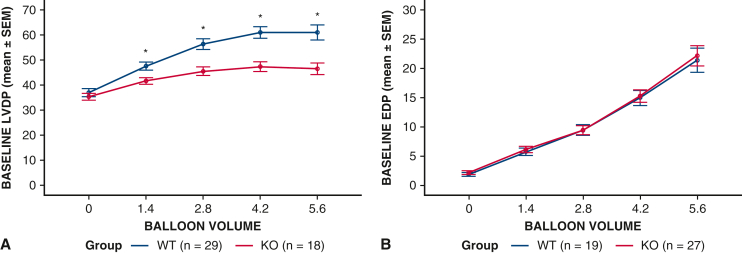
Baseline left ventricular pressures taken via intracavitary balloon at a range of balloon volumes in SUR2 (KO) and WT hearts. Isolated hearts from SUR2 KO or WT mice were cannulated and perfused with Krebs-Henseleit buffer. After 30 minutes of perfusion, baseline left ventricular pressures were measured with increasing balloon volumes. A, SUR2 KO hearts had significantly lower baseline LVDPs compared with WT. B, There was no difference in baseline EDPs between groups. ∗Simple comparisons for baseline LVDP between WT and SUR2 KO hearts were significant (*P* < .05) at all balloon volumes beyond zero using independent-samples t tests. *SUR*, Sulfonylurea receptor; *KO*, knockout; *WT*, wild type; *LVDP*, left ventricular developed pressure; *EDP*, end-diastolic pressure.

Baseline (before ischemia) LVDP had a significant main effect of group, such that it was lower in SUR2 KO hearts compared with WT regardless of balloon size (*P* < .001) (Figure 3, *A*). Because baseline differences were noted, we compared data (both LVDP and EDP) that were adjusted for baseline pressure in each heart by using change from baseline values.

**Figure 4:**
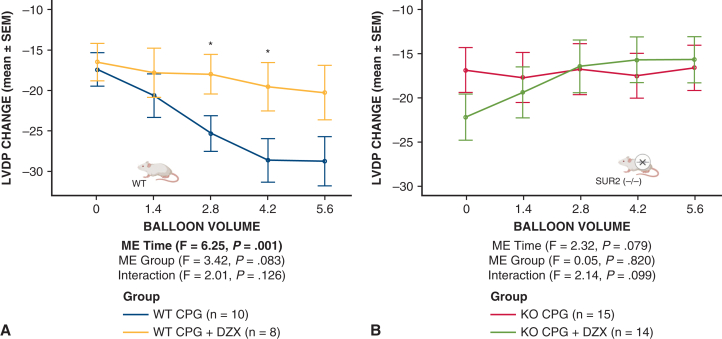
Prolonged global ischemia with hyperkalemic CPG is associated with reduced left ventricular developed pressure that is prevented by K_ATP_ channel opener diazoxide in WT but not SUR2 KO mouse hearts. Isolated mouse hearts from WT (A) or SUR2 KO (B) were subjected to 90 minutes of global ischemia after hyperkalemic cardioplegia ± DZX. Mean ± SEM change in LVDP from baseline across all LV balloon volumes after ischemia was measured and compared between groups. “ME time” is the main effect of balloon volume (regardless of group). “ME group” is the main effect of mouse type groups (collapsed across balloon volumes). “Interaction” is the interaction of balloon volume and group to indicate if the pattern of results over different balloon volumes differed by group. ∗Simple comparisons between LVDP CPG versus CPG + DZX were significant (*P* < .05) at balloon volumes 2.8 and 4.2 in WT using independent-samples t tests. *CPG*, Cardioplegia; *K_ATP_*, adenosine triphosphate-sensitive potassium; *WT*, wild type; *SUR*, sulfonylurea receptor; *KO*, knockout; *DZX*, diazoxide; *SEM*, standard error of the mean; *LVDP*, left ventricular developed pressure; *ME*, main effect; *F*, analysis of variance F test statistic.

WT hearts demonstrated decreased LVDP (relative to baseline) after global ischemia that was prevented by the addition of DZX (Figure 4, *A*). In contrast, SUR2 KO hearts demonstrated decreased LVDP (relative to baseline) that was not responsive to DZX (Figure 4, *B*).

#### End-diastolic pressure

**Figure 5:**
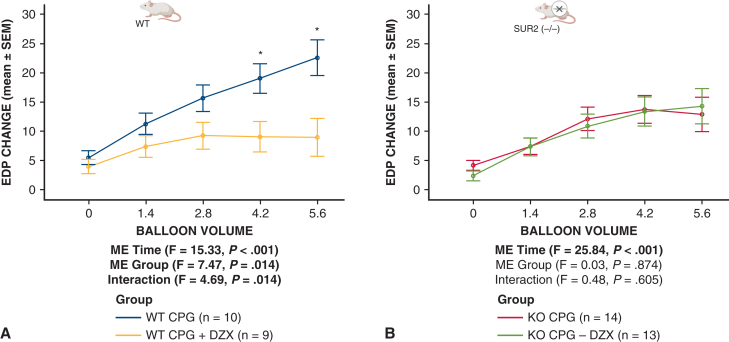
Prolonged global ischemia with hyperkalemic CPG is associated with increased EDP that is prevented by K_ATP_ channel opener diazoxide in WT but not SUR2 KO mouse hearts. Isolated mouse hearts from WT (A) or SUR2 KO (B) were subjected to 90 minutes of global ischemia after hyperkalemic CPG ± DZX. Mean ± SEM change in EDP from baseline across all balloon volumes after ischemia was measured and compared between groups. “ME time” is the main effect of balloon volume (regardless of group). “ME group” is the main effect of mouse type groups (collapsed across balloon volumes). “Interaction” is the interaction of balloon volume and group to indicate if the pattern of results over different balloon volumes differed by group. ∗Simple comparisons between EDP CPG versus CPG + DZX were significant (*P* < .05) at balloon volumes 4.2 and 5.6 in WT using independent-samples t tests. *CPG*, Cardioplegia; *EDP*, end-diastolic pressure; *K_ATP_*, adenosine triphosphate-sensitive potassium; *WT*, wild type; *SUR*, sulfonylurea receptor; *KO*, knockout; *DZX*, diazoxide; *SEM*, standard error of the mean; *ME*, main effect; *DZX*, diazoxide; *F*, analysis of variance F test statistic.

Baseline EDP was not different between SUR2 KO and WT hearts across all balloon volumes (*P* = .77) (Figure 3, *B*). Change in LV EDP after prolonged global ischemia in WT and SUR2 KO hearts is displayed in Figure 4. WT and SUR2 KO hearts demonstrated similarly increased EDP (relative to baseline) after global ischemia, but this increase was only prevented by the addition of DZX in WT hearts (Figure 5).

### Coronary Flow

**Figure 6:**
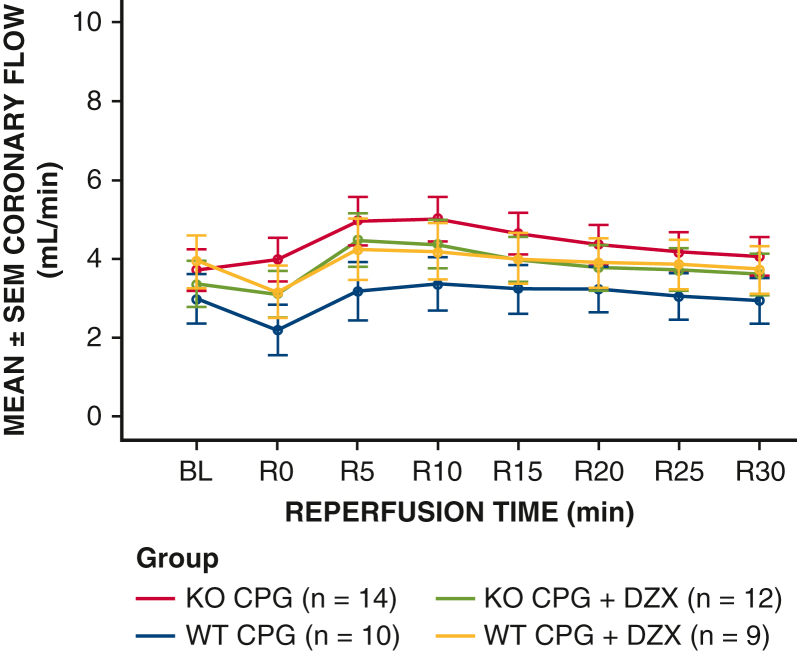
Inline coronary flow after prolonged global ischemia in SUR2 (KO) and WT mouse hearts. Isolated hearts from SUR2 KO or WT hearts were cannulated and perfused with Krebs-Henseleit buffer at 37 °C. Hearts were then subjected to 90 minutes of global ischemia after hyperkalemic cardioplegia ± DZX. Coronary flow (mL/min ± SEM) was measured by inline probe in the column. The main effect of group from the repeated measures analysis of variance was not significant (F = 0.93, *P* = .435), indicating no difference in coronary flow between groups regardless of time point (baseline and during reperfusion). *SUR*, Sulfonylurea receptor; *KO*, knockout; *WT*, wild type; *DZX*, diazoxide; *SEM*, standard error of the mean; *F*, analysis of variance F test statistic.

Coronary flow rates were not different between WT and SUR2 KO groups at any time point (baseline or reperfusion; *P* = .435) (Figure 6).

## Discussion

Previous extensive assessment of the potential for distinct K_ATP_ channel subunits to mediate DZX cardioprotection using subunit knockout models failed to implicate Kir1.1, Kir6.1, Kir6.2, and SUR1 (Table 1).^10^^,^^16^, ^17^, ^18^, ^19^, ^20^, ^21^, ^22^ Strikingly, the present study uniquely implicates SUR2 as a first K_ATP_ channel subunit in cardioprotection in a prolonged global ischemia model.

K_ATP_ channels in cardiac myocytes are composed of Kir6.2 and SUR2A subunits, whereas K_ATP_ channels in vascular smooth muscle are composed of Kir6.1 and SUR2B subunits.^32^ SUR2 KO animals lack expression of both major splice variants of the gene, SUR2A and SUR2B, and accordingly have been shown to have both cardiac and vascular abnormalities, including hypertension, coronary vasospasm (with ST elevation), arrhythmias, cardiac hypertrophy, dilated cardiomyopathy, and sudden death.^33^, ^34^, ^35^

In the current study, baseline LVDP was lower in SUR2 KO mice versus WT, and baseline LVEDP was not different between SUR2 KO and WT. This finding may suggest that the loss of the SUR2 subunit may affect contractility without affecting lusitropy or relaxation. However, we would expect baseline parameters to be unaffected by SUR2 KO because K_ATP_ channel activity is not expected under metabolically challenged conditions. This finding requires further investigation, because others have noted conflicting results, including similar baseline systolic and diastolic pressures when comparing control mice with SUR2 KO mice,^33^ a reduction in cardiac performance at baseline in SUR2 KO mice in the ventricular myocardium,^28^^,^^36^ and the fact that zebrafish with SUR2 KO demonstrate reduced baseline LVEF and no difference in LVEDV.^28^

Previous studies suggest that SUR2 KO mice may be more resistant to ischemia compared with WT mice.^33^^,^^36^ Aubert and colleagues^36^ noted significantly greater fractional recovery of LVDP in SUR2 KO hearts after ischemia compared with WT hearts (despite lower baseline LVDP), consistent with the expectation that cardiac K_ATP_ activity should be very low at baseline, but normally activated during ischemia.^37^ They also suggested that SUR2 KO (deleted exons 14-18) mice may have a preconditioned myocardium that was more resistant to stress secondary to continual vasospasm,^36^ concluding that SUR2 KO was cardioprotective, noting smaller ventricular infarct size after ischemia. Stoller and colleagues^33^ also noted that SUR2 KO hearts were resistant to ischemia in Langendorff models, demonstrating reduced infarct size and preserved LVDP after 40 minutes of global ischemia when compared with WT. We found that LVDP and EDP were relatively preserved in SUR2 KO hearts after ischemia with cardioplegia compared with baseline; however, there was no benefit noted with DZX. Of note, our model (90 minutes of global ischemia with one dose cardioplegia with or without DZX) is not an infarct model (30-40 minutes of global ischemia); thus, comparisons are complex. Plasma membrane SUR2 K_ATP_ channels may not be required for cardioprotection, as we have noted that DZX provides cardioprotection but does not open sarcolemmal K_ATP_ channels.^10^ However, we suggest that a nonplasma membrane K_ATP_ channel or alternative nonchannel mechanism involved in DZX cardioprotection may require the SUR2 subunit.

The current study suggests that the SUR2 subunit is involved in DZX cardioprotection in this model. The identification of a targeted K_ATP_ channel opener involved in myocardial protection will allow for the use of cardiac-specific agents during global ischemia during cardiac surgery.

### Limitations

Genetic disruption may result in unknown and unintended consequences, such as compensation by alterations in other channels or atypical channel formation that may affect results.^34^^,^^38^^,^^39^ For example, baseline LVDP was lower in SUR2 KO hearts compared with WT, which may reflect compensatory changes or atypical channel behavior. Because of this baseline difference, we compared groups using change from baseline values. However, genetic disruption provides a definitive method to probe for DZX cardioprotection, in contrast to nonspecific pharmacologic channel inhibition.^19^,^21^^,^^40^^,^E1, E2, E3 Although previous studies indicate that SUR2 KO hearts experience less of an insult after global ischemia, our results clearly document injury, including reduced LVDP and increased LVEDP.

The finding of consistent and equal coronary flow between all groups confirms the consistency of flow in the Langendorff column in all experiments as well as the competency of the aortic valve, thus ensuring accurate LV balloon readings. Although this model was designed to be translational, caution must be taken in extrapolation to clinical situations.

## Conclusions

This study in a global ischemia isolated heart model indicates the involvement of the SUR2 subunit in cardioprotection by the K_ATP_ channel opener DZX. This model^7^ was designed to mimic the clinical situation of global arrest during cardiac surgery. SUR2 is the first K_ATP_ channel subunit suggested to be involved in DZX cardioprotection. Identification of subunits of a cardioprotective K_ATP_ channel would allow targeted pharmacologic therapy to reduce myocardial stunning after global ischemia during cardiac surgery.^15^

## Conflict of Interest Statement

The authors reported no conflicts of interest.

The *Journal* policy requires editors and reviewers to disclose conflicts of interest and to decline handling or reviewing manuscripts for which they may have a conflict of interest. The editors and reviewers of this article have no conflicts of interest.
